# Virtual reality relaxation for people with mental health conditions: a systematic review

**DOI:** 10.1007/s00127-022-02417-5

**Published:** 2023-01-20

**Authors:** Simon Riches, Priyanga Jeyarajaguru, Lawson Taylor, Carolina Fialho, Jordan Little, Lava Ahmed, Aileen O’Brien, Catheleine van Driel, Wim Veling, Lucia Valmaggia

**Affiliations:** 1 Department of Psychology, King’s College London, Institute of Psychiatry, Psychology and Neuroscience, London, SE5 8AF UK; 2 Social Genetic and Developmental Psychiatry Centre, King’s College London, Institute of Psychiatry, Psychology and Neuroscience, London, SE5 8AF UK; 3 South London and Maudsley NHS Foundation Trust, London, BR3 3BX UK; 4 Department of Psychosis Studies, Institute of Psychiatry, Psychology and Neuroscience, King’s College London, London, SE5 8AF UK; 5 St. George’s University of London, London, SW17 ORE UK; 6 University Center for Psychiatry, University of Groningen, University Medical Center Groningen, PO Box 30.001 (HPC CC60), 9700 RB Groningen, The Netherlands

**Keywords:** Extended reality, Virtual environment, Wellbeing, Stress management, Psychological interventions, Psychiatric conditions

## Abstract

**Purpose:**

Vulnerability to stress is linked to poor mental health. Stress management interventions for people with mental health conditions are numerous but they are difficult to implement and have limited effectiveness in this population. Virtual reality (VR) relaxation is an innovative intervention that aims to reduce stress. This review aimed to synthesize evidence of VR relaxation for people with mental health conditions (PROSPERO 269405).

**Methods:**

Embase, Medline, PsycInfo, and Web of Science were searched until 17th September 2021. The review was carried out according to Preferred Reporting Items for Systematic Reviews and Meta-Analyses. The Effective Public Health Practice Project (EPHPP) tool assessed methodological quality of studies.

**Results:**

Searching identified 4550 studies. Eighteen studies (*N* = 848) were included in the review. Studies were published between 2008 and 2021. Eleven were conducted in Europe. Thirteen studies were controlled trials. Participants were mostly working-age adult outpatients experiencing anxiety or stress-related conditions. Other conditions included eating disorders, depression, bipolar disorder, and psychosis. Five studies tested inpatients. All studies used a range of nature-based virtual environments, such as forests, islands, mountains, lakes, waterfalls, and most commonly, beaches to promote relaxation. Studies provided evidence of the feasibility, acceptability, and short-term effectiveness of VR relaxation to increase relaxation and reduce stress. EPHPP ratings were ‘strong’ (*N* = 11), ‘moderate’ (*N* = 4), and ‘weak’ (*N* = 3).

**Conclusions:**

VR relaxation has potential as a low-intensity intervention to promote relaxation and reduce stress for adults with mental health conditions, especially anxiety and stress-related problems. Further research is warranted on this promising intervention.

**Supplementary Information:**

The online version contains supplementary material available at 10.1007/s00127-022-02417-5.

## Introduction

Vulnerability to stress is linked to mental health conditions, such as depression and anxiety, and has been exacerbated by the COVID-19 pandemic [1, 2]. Having a mental health condition can in turn increase stress levels, consequently creating a vicious bidirectional relationship in which stress maintains and worsens mental health symptoms [3], which can be a trigger for more severe and prolonged problems [4, 5]. Numerous stress management interventions aim to reduce stress and improve well-being [6]. Stress management techniques such as meditation, yoga, and progressive muscle relaxation can improve mental health outcomes by promoting relaxation, reducing tension, and activating the parasympathetic nervous system [7], which reduces stress-associated physiological responses, such as elevated blood pressure and heart rate [6]; while mindfulness-based interventions can improve short-term cognitive outcomes and emotion regulation [8]. Stress management relaxation techniques are widely considered to be cost-effective and low risk [9]; however, they can be difficult to implement due to time constraints, stigma about help-seeking, lack of consumer confidence, and needing high levels of consumer effort in terms of concentration and attention, which can often be impaired in people with mental health conditions [10].

Innovative approaches are therefore needed to supplement existing stress management techniques, particularly for people with mental health conditions. Virtual reality (VR) relaxation is one such innovation that is attempting to meet this need. VR is an immersive technology that is increasingly being used in various healthcare settings [11], including for people with mental health conditions [12–14]. Research indicates that VR-based relaxation, using head mounted displays (HMD) to deliver the VR, is feasible, acceptable, and is effective in the short-term to promote relaxation and reduce stress for the general, non-clinical population [15]. However, despite the emergence of promising studies that test VR relaxation in clinical samples, there is no systematic review of VR relaxation for people with mental health conditions. Therefore, the aim of this systematic review is to identify, narratively synthesize, and quality rate existing research on VR relaxation for people with mental health conditions (PROSPERO: 269405).

## Methods

### Search strategy

This review was carried out according to Preferred Reporting Items for Systematic Reviews and Meta-Analyses (PRISMA) [16]. Academic databases Embase, Medline, PsycInfo and Web of Science were searched until 17th September 2021. Search terms were: “virtual real*” OR “virtual-real*” OR “VR” OR “virtual enviro*” OR “virtual character*” OR “VCs” OR “avatar*” AND “relax*” OR “autogen*” OR “meditat*” OR “mindful*” OR “rest*” OR “PMR” OR “progressive muscle” OR “imagery” OR “breath*” OR “distract*” OR “wellness” OR “wellbeing” OR “well-being” AND “mental health” OR “mental illness” OR “mental disorder” OR “psych*" OR "schiz*" OR "mood" OR “depress*” OR “bipolar” OR “anxi*” OR “panic disorder” OR “obsessive compulsive” OR “obsessive–compulsive” OR “OCD” OR “stress” OR “PTSD” OR “dissociat*” OR “eating” OR “anorexi*” OR “bulim*” OR “substance us*” OR “substance misuse” OR “addict*” OR “dissocial” OR “personality disorder”. Truncations were used to account for alternative spellings and word endings. Search terms associated with VR and relaxation were adapted from a previous systematic review on VR relaxation for the general population [15]. Search terms associated with mental health conditions were adapted from diagnostic labels in the International Statistical Classification of Diseases and Related Health Problems, (11th ed; ICD-11; World Health Organization [17]. Studies categorized under key subject headings (‘Virtual Reality’, ‘Relaxation’, and ‘Mental Disorders’) were extracted using the ‘explode’ function on Embase, Medline and PsycInfo. These studies, and those from other sources, such as reference lists of review articles, were pooled with those identified using search terms. Studies were extracted and screened using reference management software Endnote. All stages of the search strategy and data extraction were carried out by two independent researchers (PJ, LT), under the supervision of the lead author (SR). There was regular checking and consultation on this process between the research team to ensure that relevant articles were not overlooked.

Studies were included in the review if they were published in a peer-reviewed journal; written in English; used quantitative research methods of any study design; had a sample size of *N* ≥ 5; tested clinical populations with either a formal mental health diagnosis from manuals such as ICD or DSM or, more informally, reported symptoms that could be interpreted as consistent with recognized mental health conditions; tested a virtual reality-based relaxation intervention; and measured relaxation or relaxation-related variables. Studies were excluded if they were abstracts, conference proceedings, dissertations, non-empirical, reviews, used only qualitative methods, or targeted specific, non-clinical anxieties, for example, dental anxiety or exam anxiety. The review used a narrative approach to synthesize findings. Findings were analyzed in terms of feasibility (i.e., safety, accessibility), acceptability (i.e., user experience, lack of adverse effects), and effectiveness (i.e., outcomes, impact on mental health symptoms). In the event of any discrepancies or disagreement between researchers on the search strategy and data extraction, studies were discussed between the research team (SR, PJ, LT) until discrepancies were resolved.

### Quality assessment

Quality rating was carried out by four independent reviewers (PJ, LT, CF, JL), under the supervision of the lead author (SR), using the Effective Public Health Practice Project (EPHPP) tool. EPHPP has good content and construct validity, and inter-rater reliability [18], and can provide consistent quality ratings for a range of study designs. EPHPP’s six subscales (selection bias, study design, confounders, blinding, data collection methods, and withdrawals and drop-outs) are given a rating of ‘strong’, ‘moderate’, or ‘weak’. A global rating for each study is calculated. Studies receive a global rating of ‘strong’ if there are no weak subscale ratings, ‘moderate’ if there is one weak subscale rating, and ‘weak’ if there are two or more weak subscale ratings. EPHPP reclassifies randomized controlled trials (RCTs) as controlled clinical trials (CCTs) if studies do not report information on method of randomization. Discrepancies in ratings were discussed between researchers (SR, PJ, LT, CF, JL) and studies were re-evaluated until consensus was reached.

## Results

### Study characteristics

**Fig. 1:**
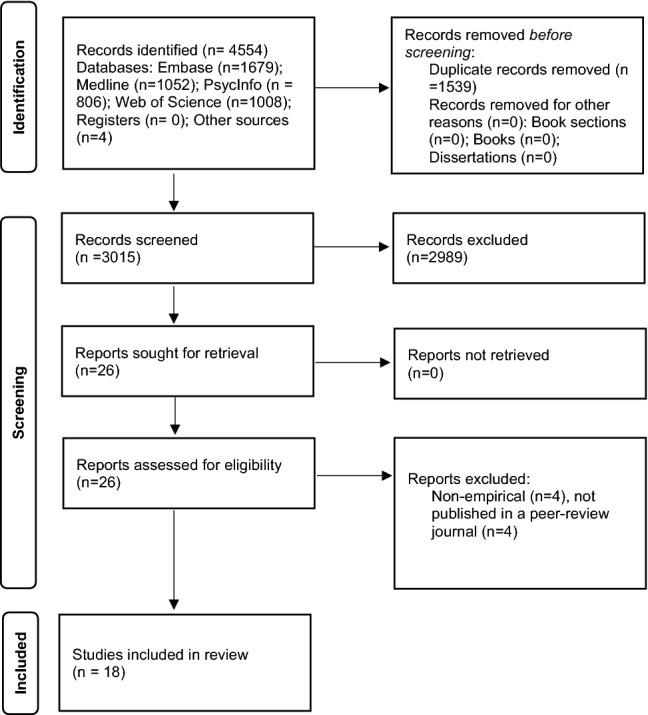
Preferred Reporting Items for Systematic Reviews and Meta-Analyses flow diagram of studies investigating virtual reality relaxation for people with mental health conditions

A total of 4,550 studies were identified through database searching and an additional four studies were identified from other sources. From this total, 26 full papers were screened, and of these, eighteen studies, published between 2008 and 2021, met inclusion criteria and were included in the review. See Fig. 1 for the PRISMA diagram. See Table 1 for full details of study characteristics. Most of the eighteen studies were from European research groups (*N* = 11); specifically, studies were conducted in Italy (*N* = 6), The Netherlands (*N* = 3), Singapore (*N* = 2), Australia (*N* = 1), Canada (*N* = 1), France (*N* = 1), South Korea (*N* = 1), Taiwan (*N* = 1), United Kingdom (*N* = 1), and United States of America (*N* = 1). In total, 848 participants took part in the studies. Almost all studies tested working-age adult participants, who were mostly female. There were four RCTs and nine CCTs. Study samples ranged from eight to 175 participants; seven studies had samples of over 50 participants; and two studies used the same sample due to the participants being followed up three months after the initial study [19, 20]. Thirteen VR interventions had interactive components for participants to engage with and five were passively viewed by participants. Sixteen interventions were facilitator-led, one was self-led by participants in their homes, and in one case, this aspect of the intervention was unclear.

**Table 1:** Characteristics of studies of virtual reality relaxation for people with mental health conditions

Study	Country	Participants	*N*	Mean age (SD)	Equipment	Virtual Environment	Measures	Sessions	Intervention	Findings
Bossenbroek et al. [35] (2020)	The Netherlands	Outpatients at a specialist secondary school for adolescents with psychiatric and behavioral problems, including ADHD or ASD, with either ED, ODD, PD, RAD or SAD	8 (1 female, 7 males); one group	14.67 (1.83)	HTC VIVE HMD with headphones; DEEP VR biofeedback game; DEEP breathing belt with Arduino- FLORA wearable electronic platform for movement in virtual environment. Arduino Software	Underwater fantasy world. No other information provided	STAI, disruptive classroom behavior measured by teachers using Likert Scale	6 (daily, for 12.41 min)	DEEP VR biofeedback game. Interactive intervention and facilitator-led	Participants showed reductions in state anxiety and disruptive behaviors
Gorini et al. [22] (2010)	Italy	Outpatients with Generalized Anxiety Disorder	20 (no gender data reported); three groups: VR and mobile phone with biofeedback (*N* = 4), VR & mobile phone without biofeedback (*N* = 8), controls (*N* = 8)	Not reported	HMD with head tracking but no further information. 3DVIA Virtools 4.1 software; HTC Touch Pro	Tropical island with campfire, beach, waterfall, and gazebo (with words/images about stressful situations). Audio narrative of relaxation techniques such as muscle relaxation	BAI, HAM-A, PSWQ, STAI-Y, GSR/HR sensor module	8	VR/biofeedback group’s HR variations modified virtual environment e.g., changed fire intensity, movement of waterfall, etc. VR group without biofeedback experienced VR. Control group did not receive VR. Interactive intervention and facilitator-led sessions with self-led mobile phone element for use at home	Both VR groups decreased in anxiety. Biofeedback group decreased in state anxiety. Non-biofeedback group and control group decreased in worry
Habak et al. [32] (2021)	Australia	Outpatients with depression or suicidality	79 (53 females, 23 males, 3 non-binary)	Most common age range: 25–34. Full Age Range: 18–65 +	Edge of the Present mixed reality environment software. No HMD information	Rainforest, tropical beaches, desert, with environmental effects to intensify sensory experience (e.g., a warm breeze)	BHS, PANAS, Sense of Presence, SWEMWBS, VR feedback	1 (10 min)	Participants explored scenes freely. Interactive but does not state whether facilitator or self-led	Wellbeing and positive mood increased. Negative mood and hopelessness decreased. Sense of presence was very high
Kim et al. [28] (2021)	South Korea	Outpatients with high stress	74 (37 females, 37 males); two groups: VR relaxation first (*N* = 36), biofeedback first (*N* = 38)	Mean and SD not reported. Median age = 39, Range = 19–59	Samsung Gear VR, head tracking, stereo earphones, ProComp Infiniti Biofeedback system	Relaxing video: natural scenes on trekking course with relaxing soundtrack. Stress video: walking on a shaky (moving) path	PSS-10, STAI-X1, STAI-X2, NRS, PANAS, SDS, EQ-5D-5L, SSQ, HRV	2 (1 for each relaxation type, approximately 28 min each)	All participants were presented with the stress video and then asked to perform a mathematical task. Then the VR relaxation first group watched the relaxing video and the biofeedback first group carried out relaxation techniques while viewing their own biofeedback data on a screen. The following day, the groups swapped exercises. Passive intervention and facilitator-led	Both VR and biofeedback interventions reduced stress. There were no significant differences between interventions
Maarsingh et al. [29] (2019)	The Netherlands	Outpatients who experience significant stress and healthy controls	175; two groups: Patients (*N* = 64, 52% females, 48% males), healthy controls (*N* = 111, 62% female, 38% male)	No age data for total sample. Patients: 40.6 (11.5). Healthy controls: 43.0 (10.5)	HTC Vive HMD, controllers, computer. Stressjam game	Tropical jungle island with temples and other buildings	SMM-G, HRV	3 (1 h)	Stressjam game challenged participants to overcome increasingly difficult obstacles (e.g., climbing a rope) by increasing or decreasing their stress levels. Interactive intervention and facilitator-led	Patients experienced stress in a more functional way post-intervention
Malbos et al. [21] (2020)	France	Outpatients with Generalized Anxiety Disorder	27 (13 females, 14 males); two groups: VR relaxation therapy, mental imagery relaxation therapy. Number of participants in each group not reported	48.40 (11.91)	Sensics zSight HMD and head tracker; remote control with a directional pad; CryEngine Sandbox (Crytek GmbH) software	Tropical beach, campfire in forest, polar ice fields, living room, journey across the solar system, mountain peak in clouds. Audio track of flowing water, birds, choices of music, etc.	BDI-II, PSQW, SF-12 Quality of Life Questionnaire, STAI Y-A, STAI Y-B, SUD, HR, PQ, SSQ	6 (30 min weekly)	VR group selected virtual environment and music. Participants were able to explore the environments by walking, swimming, and interacting with 3D objects (e.g., opening doors). Sessions included relaxation techniques. Imagery group combined relaxation and mental imagery techniques. Interactive intervention and facilitator-led	Both groups decreased in anxiety, worry and depression, and increased in quality of life
Manzoni et al. [19] (2008)	Italy	Inpatients with emotional eating in the context of obesity	60 (all females); three groups: VR, imaginative, controls (all *N* = 20)	No age data for total sample. VR group = 42.80 (11.44), imaginative group = 48.55 (7.96), control group = 39.65 (14.52)	Sony Glasstron PLM S-700 HMD, position tracker, earphones, joystick, NeuroVR 1.5 software, Asus G2S laptop	Relaxing environment: green valley with lake and mountain, relaxing narrative/ audio track, e.g., birds, water, etc. Stressful environments: kitchen, restaurant, supermarket, office, etc	BDI, WELSQ, STAI, relaxation VAS, HR	12 (1 h, 4 per week, for 3 weeks)	Both VR and imaginative groups learned relaxation techniques. VR group then carried them out in VR in both relaxing and stressful environments. Imaginative group carried them out with relaxing audio track only and imagined both relaxing and stressful situations. Interactive intervention and facilitator-led. The control group received treatment as usual	VR and imaginative groups increased in relaxation and self-efficacy about eating control and decreased in depression, anxiety, and heart rate. All groups decreased in weight
Manzoni et al. [20] (2009)	Italy	Inpatients with emotional eating in the context of obesity	36 (all females, follow-up subsample of Manzoni et al. 2008). Three groups: VR (*N* = 12), imaginative (N = 14), controls (*N* = 10)	Not reported	Sony Glasstron PLM S-700 HMD, position tracker, earphones, joystick, NeuroVR 1.5 software, Asus G2S laptop	Relaxing environment: green valley with lake and mountain, relaxing narrative/audio track, e.g., birds, water, etc. Stressful environments: kitchen, restaurant, supermarket, office, etc	STAI, EOQ, BDI, WELSQ three months after intervention	12 (1 h, 4 per week, for 3 weeks)	Both VR and imaginative groups learned relaxation techniques. VR group then carried them out in VR in both relaxing and stressful environments. Imaginative group carried them out with relaxing audio track only and imagined both relaxing and stressful situations. Interactive intervention and facilitator-led. The control group received treatment as usual	VR and imaginative groups increased in self-efficacy about eating control and decreased in depression, anxiety, and emotional eating. VR group were significantly lower in emotional eating than imaginative group. All groups maintained or decreased in weight
Mark et al. [33] (2021)	United Kingdom	Psychiatric Intensive Care Unit (PICU) inpatients with Psychosis	17 (all males)	35.8 (9.63)	Oculus Go HMD. No software information reported	Videos of forests, beach, swimming with dolphins or turtles, watching animals (e.g., elephant orphanage, African safari), climbing trees, space ride, hot air balloon ride	Qualitative observations of distress, mental state, risk, VR experience	1 session (3–10 min)	Participants watched one or two videos of their choice. Passive intervention and facilitator-led	All participants found the VR acceptable and reported it was a positive experience. Sixteen participants reported they would use VR again. Two participants reported they did not trust/feared VR
Mistry et al. [27] (2020)	Canada	Outpatients with PTSD	96 (54 females, 42 males). Clinical sample (*N* = 26) Eight groups with varying VR and non-VR meditations (all *N* = 12)	Not reported	Dell Visor Windows Mixed Reality HMD, hand controllers, headphones, Microsoft Windows 10 computer and Mixed Reality functionality powered the VR Guided Meditation application. No software information reported	Tropical rainforest, hidden cave, island, underwater coral reef, foreign planet	LES, LEC-5, ACE, PCL-5, TRASC, mDES, BASS, MEQ, Satisfaction and Credibility Questionnaire, verbal feedback	1 (2 guided meditations, each lasted approximately 5 min)	Participants completed VR and non-VR meditations in varying orders. In the non-VR meditation, participants watched the virtual environment on a screen or closed their eyes. Interactive intervention and facilitator-led	Participants who completed the VR meditation first reported greater positive affect. Most participants preferred VR meditation to non-VR meditation. Clinical sample reported increased distress in both VR and non-VR compared to healthy sample
Pallavicini et al. [23] (2009)	Italy	Outpatients with Generalized Anxiety Disorder	12 (9 females, 3 males); three groups: VR and mobile phone with biofeedback (*N* = 4), VR and mobile phone without feedback (*N* = 4), controls (*N* = 4)	No age data for total sample. VR/biofeedback group = 41.25 (13.24), VR without biofeedback group = 48.5 (12.662), controls = 51.25 (9.845)	Vuzix iWear HMD, Asus G2S computer, therapist’s netbook EEPC 100H – BK039X for controlling virtual environment, joystick, Virtools software, GSR/HR Sensor Module, HTC Touch Pro T7272	Tropical island with campfire, beach, waterfall, and gazebo (with words/images about stressful situations). Audio narrative of relaxation techniques such as muscle relaxation	GAD-7, PSWQ, BAI, STAI-Y2, HAM-A, STAI Y-1, VAS measuring anxiety, GSR, HR	8	VR/biofeedback group’s HR variations modified virtual environment, e.g., changed fire intensity, movement of waterfall, etc. VR group without biofeedback experienced VR. Interactive intervention and facilitator-led sessions with self-led mobile phone element for use at home. Control group did not receive VR	Both VR groups had a greater decrease in heart rate, anxiety, and worry compared to controls
Repetto et al. [24] (2013)	Italy	Outpatients with Generalized Anxiety Disorder	25 (16 females, 9 males); three groups: VR and mobile group with biofeedback (*N* = 7), VR and Mobile group without biofeedback (*N* = 9), controls (*N* = 8)	No age data for total sample. VR/biofeedback group = 45.25 (14.24), VR without biofeedback group = 48.5 (12.662), controls = 49.25 (9.845)	Vuzix iWear HMD, Asus G2S computer, therapist’s netbook EEPC 100H – BK039X for controlling virtual environment, joystick, Virtools software, GSR/HR Sensor Module, HTC Touch Pro T7272	Tropical island with campfire, beach, waterfall, and gazebo (with words/images about stressful situations). Audio narrative of relaxation techniques such as muscle relaxation	BAI, STAI-Y2, HAM-A, STAI-Y1, HR, GSR	8	VR/biofeedback group’s HR variations modified virtual environment, e.g., changed fire intensity, movement of waterfall, etc. VR group without biofeedback experienced VR. Interactive intervention and facilitator-led sessions with self-led mobile phone element for use at home. Control group did not receive VR	Both VR groups decreased in heart rate and anxiety
Riva et al. [30] (2008)	Italy	Outpatients with high anxiety in the context of obesity and history of emotional eating	40 (all females); three groups: VR stress management (*N* = 15), DVD stress management (*N* = 11), no treatment (*N* = 14)	Not reported	Not reported	Tropical island with different zones e.g., watching waves, barrier reef. Audio narrative of relaxation techniques	ITC-SOPI, PANAS, STAI, VAS of emotional states	2 (1 h, on consecutive days)	Stress management protocol included imagery, relaxation and different cognitive behavioral approaches. Identical script and exercises both for VR and DVD but different imagery. Passive intervention and facilitator-led	VR group had a significantly greater reduction in anxiety compared to other groups. Anxiety reduction and increase in positive emotion were associated with presence
Shah et al. [31] (2015)	Singapore	Inpatients with a diagnosis of Major Depressive Disorder and Bipolar Disorder (with depressive episode)	22 (16 females, 6 males)	Mean and SD not reported. Range: 21–60	ITG-PCX3 HMD, VR DE-STRESS program	Beach, auditory instructions, relaxing music	DASS-21, PRS, KSSMQ, BP, HR, ST, feedback form	3 (1 h, for 3 days)	Psychoeducation on causes, symptoms and management of stress; mood disorders, and the relationship with stress. VR with relaxation techniques. Interactive intervention and facilitator-led	Post-VR, participants reported significantly lower stress, depression and anxiety, and greater relaxation and knowledge. Participants reported the intervention was beneficial as it allowed them to think positively, learn to relax and manage stress
Tan et al. [36] (2021)	Singapore	In-patients with mild or moderate mental disorder e.g., schizophrenia, bipolar disorder, mood disorders	40 (24 females, 16 males); two groups: VR (*N* = 19) and controls (*N* = 21)	No age data reported	iTVGoggles Wide View 3D. No software information reported	Bhutan scenery, e.g., mountains, forests, skies, rivers. Japanese scenery. Hot air balloons accompanied with soothing music	NSRS, HR, BP, ST, Perceived Relaxation Scale, PSS-10, KSMM, participant feedback	2 (40 min)	Psychoeducation on stress. VR with breathing and muscle relaxation. Passive intervention and facilitator-led	Post-VR, VR group reported significantly greater relaxation and reduced stress
Tarrant et al. [25] (2018)	United States of America	Outpatients with moderate level of Generalized Anxiety Disorder	26 (20 females, 6 males); two groups: VR (*N* = 14) and controls (*N* = 12)	No age data for total sample. VR group = 46.21 (10.77), controls = 48.17 (20.11)	Gear VR HMD powered by Samsung S7, Mindfulness in nature experience, by StoryUp VR	Mountain landscape, rocks, blue skies, with soft music and female voice to guide mindfulness exercises	GAD-7, EEG, STAI	1 (75 min)	All groups rested. Then the VR group experienced VR. Control group experienced further rest. Passive intervention and facilitator-led	Both VR and control groups decreased in state anxiety. VR group showed significant electrophysiological markers demonstrating lower anxiety
Veling et al. [34] (2021)	The Netherlands	Outpatients with a diagnosis of anxiety, psychotic, depressive or bipolar disorder	50 (33 females, 17 males); two groups: VR relaxation (*N* = 25), standard relaxation exercises (*N* = 25)	41.6 (14.2)	Samsung Galaxy S6 or S7 smartphone, connected to the Samsung Gear VR HMD, VRelax software	Beaches, coral reef with tropical fish, swimming with dolphins, mountain meadow with animals, drone flight over river landscape, sea view from cliff, mountain scenery in the Alps, beach session of Tibetan singing bowl therapy	BAI, GPTS, IDS, PSS-10, SSQ, VAS on positive and negative affective state	20 (minimum of 10 min, 10 consecutive days per intervention)	The VR group was able to freely explore and select VR scenes. Standard relaxation was audio tracks of guided meditation and progressive relaxation techniques. Interactive intervention and self-led in participants homes	Both groups reported significant improvements in positive affective states and significant reductions of negative affective states. VR group had a significantly greater reduction of negative affective states, including anxiety, compared to standard relaxation. There were no significant differences with stress and symptoms
Wang et al. [26] (2020)	Taiwan	Outpatients with Generalized Anxiety Disorder	77 (38 females, 39 males); two groups: virtual nature (*N* = 40), virtual abstract painting (*N* = 37)	No age data for total sample. Virtual nature group = 58.43 (7.37), virtual abstract painting group = 59.87 (6.99)	Two projectors, curved screen with 3D environment, static bike used to ‘cycle’ through virtual environments. No software information provided	Virtual nature: forests, parks, rivers, woodlands. Virtual abstract painting: colors such as blue, green, yellow	GAD-7, MMSE, EEG, HR, perceived stress, restorative quality, satisfaction scale	1 (20 min)	All participants cycle at a moderate intensity. Virtual nature groups cycle through landscapes. Virtual abstract painting group cycle through abstract paintings. Interactive intervention and facilitator-led	Both groups displayed significant improvements in restorative quality, satisfaction, and relaxation. Virtual nature group had significantly higher levels of restorative quality and satisfaction post-VR compared to the virtual abstract painting group. Only the virtual nature group had significantly reduced stress

*ACE* adverse childhood experiences, *ADHD* attention deficit hyperactivity disorder, *ASD* autism spectrum disorder, *BAI* Beck anxiety inventory, *BASS* Buddhist affective states, *BDI* Beck’s depression inventory, *BHS* Beck hopelessness scale, *DASS-21* 21-item depression anxiety stress scale, *ED* eating disorder, *EOQ* emotional overeating questionnaire, *EQ-5D-5L* five-level version of the EQ-5D (measuring quality of life, EuroQoL Group), *GAD-7* generalized anxiety disorder 7-item scale, *GPTS* green paranoid thoughts scale, *HAM-A* Hamilton anxiety rating scale, *HMD* head mounted display, *IDS* inventory of depressive symptomatology, *ITC-SOPI* ITC- sense of presence inventory, *KSMMQ* knowledge on stress and medication management questionnaire, *LEC-5* life events checklist for DSM-5, *LES* life events survey, *mDES* modified differential emotions scale, *mDES-NA* negative affect, *mDES-PA* positive affect, *MEQ* normative meditative experiences, *MMSE* mini mental state examination, *NRS* numeric rating scale, *NSRS* numeric stress rating scale, *ODD* oppositional defiant disorder, *PANAS* positive and negative affect schedule, *PCL-5* posttraumatic stress disorder checklist for DSM-5, *PD* personality disorder, *PII* personal involvement inventory, *PQ* presence questionnaire, *PRS* perceived relaxation scale, *PSS* perceived stress scale, *PSS-10* 10 item version of perceived stress scale, *PSWQ* Penn state worry questionnaire, *RAD* reactive attachment disorder, *SAD* social anxiety disorder, *SDS* Sheehan’s disability scale, *SMM-G* stress mindset measure, *SSQ* simulation sickness questionnaire, *SUS* system usability scale, *SWEMWBS* short Warwick–Edinburgh mental well-being scale, *STAI-Y* state–trait anxiety inventory, *SUD* subjective units of discomfort, *TRASC* trauma-related altered states of consciousness, *VAS* visual analog scales, *WELSQ* weight efficacy life-style questionnaire

*BP* blood pressure, *EEG* electroencephalogram, *GSR* galvanic stress response, *HR* heart rate, *HRV* heart rate variability, *IM* imaginative, *N* Number of, *SD* standard deviation, *ST* skin temperature, *TAU* treatment as usual, *VR* virtual reality, *Wl* waiting list

Fifteen studies tested samples of participants with one specific mental health condition. Six studies tested people with Generalized Anxiety Disorder [21–26]. Three studies tested people experiencing stress, such as posttraumatic stress [27], high stress [28], and stress-related problems [29]. Three studies tested people who experienced emotional eating in the context of obesity [19, 20], including with high anxiety [30]. Two studies tested people with depression, including Major Depressive Disorder and Bipolar Disorder with a depressive episode [31], and in the context of suicidality [32]. One study tested people with psychosis [33]. Three studies tested mixed samples of participants with a range of different diagnoses. One study tested participants with mental health conditions such as anxiety, bipolar disorder, depressive, and psychotic disorders, where 30% had an anxiety-related disorder [34]; another study tested mostly male adolescent participants with attention deficit hyperactivity disorder (ADHD) or autism spectrum disorder (ASD), accompanied with anxiety and behavioral problems [35]; and another tested participants with depression, bipolar disorder, or schizophrenia [36]. Thirteen studies tested outpatient service users and five studies tested inpatient service users on psychiatric wards, including one study that took place in a psychiatric intensive care unit with a male sample [33]. Most studies on outpatient participants were conducted in labs, apart from the study on adolescents with ADHD or ASD, which was conducted in a specialist school [35] and another which involved participants of various diagnoses accessing content remotely using an HMD at home [34].

Studies varied considerably in number of VR relaxation sessions offered. One study used 20 sessions of VR relaxation, two studies used 12, three studies used eight, two studies used six, two studies used three, three studies used two, and five studies used one. All studies used a range of nature-related scenes, such as forests, islands, mountains, lakes, waterfalls, and most commonly, beaches. One study involved cycling through virtual forests and parks while seated on a real-world static bike [26]; but all other studies involved participants in a stationary position, generally seated, wearing an HMD, often with relaxing narratives or audio. Six studies involved participants using biofeedback to control in-game play, on a tropical island, a trekking path, or underwater, in order for users to change features of the virtual environment, such as the movement of a waterfall or the waves of the sea, or the intensity of a campfire on a beach [22–24, 28, 29, 35]. In these biofeedback-assisted studies, biofeedback data, such as a participant’s heart rate, could be used to reduce the intensity of the virtual campfire until it disappeared or could make the virtual sea calmer. Some studies used stressful virtual environments or exercises, such walking on a virtual shaky path, words or images related to stressful events, or a mathematical task, against which to compare virtual relaxation environments [22, 28]. Seven studies incorporated standard, non-virtual relaxation techniques, such as progressive muscle relaxation [21] and guided meditation using an audio track [34], in combination with the VR relaxation. One study reported follow-up sessions, which occurred three months after patients were discharged from hospital following treatment for emotional eating [20]. HMDs used in the studies were Samsung Gear (*N* = 3), HTC Vive (*N* = 2), Sony Glasstron (*N* = 2), Vuzix iWear (*N* = 2), Dell Visor Windows (*N* = 1), iTVGoggles (*N* = 1), model ITG-PCX3 (*N* = 1), Oculus Go (*N* = 1), and Sensics zSight (*N* = 1). One study used 3D wraparound projection technology, using two projectors, to display the 3D environment [26]. Three studies did not report HMD models [22, 30, 32]. Most studies used different software, and several did not provide software information.

### Evidence of feasibility, acceptability, and effectiveness

Studies used self-report scales to measure relaxation, mood, anxiety, worry, and perceived stress [21–23, 26, 34, 36]. Other measures, such as observational teacher reports, were used to measure behavioral problems [35]. Some studies used physiological measurements, such as heart rate and galvanic stress response, as indicators of stress [22, 31, 36]. All studies had findings that indicated that VR relaxation is feasible, including in non-lab-based settings, such as psychiatric wards for various mental health conditions [33, 36] and in a specialist school [35]. Studies reported that VR relaxation is accessible [25] and can reduce pressure on clinicians [27, 31]. Studies indicated that nature-based VR environments are feasible as a low-intensity treatment for people with mental health conditions [26, 34], including for people who experience severe and acute conditions [33], and especially where participants lack access or exposure to real-world nature [25]. Most studies reported no user difficulties. A small number of participants in a minority of studies sometimes observed discomfort with HMDs [19] or experienced other user difficulties [23]. One study reported participants experiencing cybersickness, the phenomenon of motion sickness experienced in VR, which resulted in two drop-outs [34]; while others indicated some visual discomfort [33] and discomfort wearing HMDs over glasses [36]. However, all studies indicated overall acceptability of VR relaxation. Participants mostly reported that VR relaxation was calming [27, 32], helpful [31], enjoyable [27], relaxing [25, 33], useful [23], and an experience that they would recommend to others [27, 36].

Studies indicated that VR relaxation led to short-term improvements in levels of relaxation, and most reported reduced anxiety or stress. In most studies that included another, non-virtual form of relaxation exercise as a control condition, this exercise was also found to improve levels of relaxation and stress. However, VR relaxation was always more effective or equally effective when compared to the non-virtual relaxation exercises. Six studies measured perceived stress and reported improvements in levels, experience, or management of stress, four of which were RCTs or CCTs. Five of these studies showed significantly greater short-term improvements in stress levels, and experience of stress, following VR relaxation [26, 28, 29, 31, 36]. Studies also found post-VR reductions in worry [21–23], hopelessness [32], and negative affect [32, 34]. Four studies reported a decrease in depression or depressive symptoms [19–21, 31]. Studies also found an increase in positive affective states [27, 32, 34], positive emotions [30], positive thinking [31], and quality of life [21]. One CCT reported lower levels of subjective anxiety in both experimental and control groups with generalized anxiety disorder; however, physiological markers confirmed more significant changes in the experimental group who experienced VR relaxation [25]. Another CCT highlighted the effectiveness of nature exposure in VR, as outpatients with generalized anxiety disorder had significantly reduced levels of stress compared to an alternative, non-nature-based environment [26]. One large CCT, with the largest sample size (*N* = 175) of all studies included in this review, found that outpatients with significant stress at baseline could experience stress in more functional ways after three sessions of VR relaxation [29]. The small cohort study (*N* = 8) on adolescents with ASD and ADHD found that a biofeedback game reduced short-term state anxiety and disruptive classroom behavior [35]. The only study reporting longer-term data was an RCT that collected follow-up data from 36 inpatient female adults with eating disorders. This study found improved self-efficacy in eating after VR relaxation, which was supported by follow-up data after three months. Reduction in trait anxiety was also observed at follow-up [20].

### Quality ratings

Eleven studies were given a global EPHPP rating of ‘strong’, four were rated ‘moderate’, and three were rated ‘weak’. See Table 2 for full-quality ratings. All ‘strong’ studies reported positive outcomes. All studies were moderate for selection bias as they were all judged to be somewhat likely to be representative of the target population. Thirteen of the eighteen studies were rated as strong for study design. In some cases, studies that described themselves as RCTs in the published paper were reclassified in the review as CCTs because the studies did not report information on method of randomization. Twelve out of eighteen studies were rated as strong for their controlling of confounding variables, and the remaining studies were weak, as little or no information was provided. All studies were rated moderate for blinding as information about whether the outcome assessor was aware of the intervention or whether the participants were aware of the research questions was not provided. For data collection methods, seven out of eighteen studies were rated strong, as they reported both validity and reliability. Twelve of the eighteen studies were rated strong for withdrawals and dropout, as 80–100% of their participants completed the study.

**Table 2:** Quality assessment using Effective Public Health Practice Project of studies of virtual reality relaxation for people with mental health conditions

Study	A. Selection bias	B. Study design	C. Confounders	D. Blinding	E. Data collection method	F. Withdrawals and drop out	Global rating
Bossenbroek et al. [35] (2020)	MODERATE	MODERATE	STRONG	MODERATE	STRONG	STRONG	STRONG
Gorini et al. [22] (2010)	MODERATE	STRONG	WEAK	MODERATE	MODERATE	WEAK	WEAK
Habak et al. [32] (2021)	MODERATE	MODERATE	STRONG	MODERATE	MODERATE	STRONG	STRONG
Kim et al. [28] (2021)	MODERATE	STRONG	WEAK	MODERATE	MODERATE	STRONG	MODERATE
Maarsingh et al. [29] (2019)	MODERATE	MODERATE	WEAK	MODERATE	STRONG	WEAK	WEAK
Malbos et al. [21] (2020)	MODERATE	STRONG	WEAK	MODERATE	MODERATE	MODERATE	MODERATE
Manzoni et al. [19] (2008)	MODERATE	STRONG	STRONG	MODERATE	MODERATE	STRONG	STRONG
Manzoni et al. [20] (2009)	MODERATE	STRONG	STRONG	MODERATE	MODERATE	MODERATE	STRONG
Mark et al. [33] (2021)	MODERATE	MODERATE	STRONG	MODERATE	WEAK	STRONG	MODERATE
Mistry et al. [27] (2020)	MODERATE	STRONG	WEAK	MODERATE	MODERATE	STRONG	MODERATE
Pallavicini et al. [23] (2009)	MODERATE	STRONG	STRONG	MODERATE	MODERATE	STRONG	STRONG
Repetto et al. [24] (2013)	MODERATE	STRONG	STRONG	MODERATE	MODERATE	STRONG	STRONG
Riva et al. [30] (2008)	MODERATE	STRONG	WEAK	MODERATE	WEAK	WEAK	WEAK
Shah et al. [31] (2015)	MODERATE	MODERATE	STRONG	MODERATE	STRONG	STRONG	STRONG
Tan et al. [36] (2021)	MODERATE	STRONG	STRONG	MODERATE	STRONG	STRONG	STRONG
Tarrant et al. [25] (2018)	MODERATE	STRONG	STRONG	MODERATE	STRONG	MODERATE	STRONG
Veling et al. [34] (2021)	MODERATE	STRONG	STRONG	MODERATE	STRONG	STRONG	STRONG
Wang et al. [26] (2020)	MODERATE	STRONG	STRONG	MODERATE	STRONG	STRONG	STRONG
Total	0 STRONG; 18 MODERATE; 0 WEAK	13 STRONG; 5 MODERATE; 0 WEAK	12 STRONG; 0 MODERATE; 6 WEAK	0 STRONG; 18 MODERATE; 0 WEAK	7 STRONG; 9 MODERATE; 2 WEAK	12 STRONG; 3 MODERATE; 3 WEAK	11 STRONG; 4 MODERATE; 3 WEAK

## Discussion

This systematic review aimed to narratively synthesize current evidence of feasibility, acceptability, and effectiveness of VR relaxation for people with mental health conditions. The review included eighteen studies; there were thirteen controlled studies, of which seven were RCTs. Seventeen studies tested adult participants, with a range of mental health conditions, mostly anxiety and stress-related; and one study tested adolescents with ASD and ADHD. All studies used nature-based VR environments and provided evidence of feasibility, acceptability, and short-term effectiveness of VR relaxation to promote relaxation and reduce stress. A minority of studies highlighted emerging evidence for the applicability of VR relaxation to support service users on psychiatric wards; and for its application to a wider range of more severe mental health conditions, such as eating disorders, depression, bipolar disorder, and psychotic disorders. This wider application is consistent with previous research that indicates that VR is a tool that can support social functioning impairments in people with more severe mental health conditions, such as psychosis [37–39].

Although some of the comparison conditions, such as guided meditation and progressive muscle relaxation, were also found to be effective in aiding relaxation and reducing symptoms of anxiety, low mood, and stress [21, 34], the general outcome was that VR relaxation was more or equally effective compared to these other, non-VR-based relaxation interventions. This indicates that VR relaxation has potential to supplement existing relaxation techniques or provide users with alternative relaxation and stress-reduction options. 

VR relaxation is a low-effort intervention in terms of attention and concentration due to its immersive nature; it has a standalone use; it has fewer requirements from staff and is less time intensive; and it can be used autonomously, even in the home. VR relaxation could be especially helpful during and after the COVID-19 pandemic, given that the pandemic has led to the increase and exacerbation of mental health problems [40]. This application may be especially timely given that VR has recently become much more affordable and more accessible in portable ways, and so this technology is likely to be available to more users than ever before [34, 41]. Nevertheless, the digital divide remains a significant problem and is highly likely to exclude people from lower socioeconomic backgrounds and various other groups who may be less likely to have access to technology, such as older adults [42]. Given that people from lower socioeconomic backgrounds are at greater risk of developing mental health problems, VR relaxation interventions may be less likely to reach this population and so this remains an ongoing challenge to make effective digital interventions more accessible [43].

### Strengths and limitations of studies included in the review

Strengths of the studies included in this review are the innovative use of technology; the commonalities in VR environments and measures of stress, anxiety, and relaxation, which enables comparison between studies; and the frequent use of control groups and standardized pre- and post-intervention measures, which meant that the overall quality of the studies was acceptable.

Limitations of the studies include the small clinical samples in several studies; lack of large RCTs; and testing mostly working-age adults, female participants, and clinical samples with mainly anxiety disorders or stress-related problems, which limits generalizability, especially to other genders, age groups, and other mental health conditions. Use of mixed diagnosis samples could be interpreted as a weakness in that it can hinder comparison between studies; but it could also be perceived as a strength because stress is a transdiagnostic factor in many mental health conditions and DSM and ICD classifications are not neatly defined in clinical practice, given the many comorbidities, overlapping symptoms, and subthreshold symptoms. Most studies used self-report measures to assess relaxation, stress, and other outcome variables, and therefore their findings could be subject to researcher or participant bias. Use of physiological outcome measures was limited, which would be one way to address this bias. A major limitation of the research is lack of evidence of longer-term benefits of VR relaxation for people with mental health conditions given that only one study collected follow-up data. This is consistent with a similar finding about short-term evidence and lack of longer-term data in non-clinical samples [15]. Other methodological issues such as lack of controlling for potential confounding variables and lack of information on randomization and blinding also weakens the research. Given the dominance of positive findings, it is also possible that the literature is subject to publication bias.

Overall, studies indicate emerging evidence for the potential of VR relaxation for mental health service users, especially to treat anxiety or stress-related problems. However, at this stage, one should be cautious about making stronger claims about its effectiveness or about its application to a wider range of mental health conditions.

### Strengths and limitations of the review

This is the first systematic review to narratively synthesize the current evidence of feasibility, acceptability, and effectiveness of VR relaxation for people with mental health conditions. The review included a comprehensive search strategy, using a combination of formal searches of four databases and non-indexed reference lists. The review used the EPHPP, which is an evidenced tool that assesses the methodological quality of quantitative studies, and was administered by independent reviewers, thus strengthening the methodological rigor of the review.

Important limitations of the review include the limited number of studies, and lack of standardization of interventions, conceptual definitions, and measures within the existing research. For instance, the concept of relaxation was interpreted and measured in the included studies using a variety of self-report surveys, physiological parameters, and qualitative observations, which lacks consistency and limits comparisons within the review. Similarly, despite the dominant use of nature-based themes, virtual environments used in the studies were not standardized, including both animated and video content; and there was great variation in HMDs and software, intervention tasks, level of interactivity, and number and duration of sessions, which also limits comparison between studies. In some cases, the interactivity component comprised participants making choices about which environments they explored, whereas other interventions included more active, gamified elements to the interactivity. Similarly, the role and level of input of facilitators was unclear and not standardized across studies. The review deliberately and necessarily employed a broad concept of mental health, including clinical samples with recognized diagnoses or conditions, symptoms consistent with a mental health diagnosis, and mixed samples of various diagnoses and conditions. This broad approach was intended to provide a comprehensive review of the clinical interventions that have been tested in what is still a relatively new field of research with a limited number and range of studies, but it could be interpreted as a limitation, and the broad scope of the review should be considered when interpreting the findings.

Lack of inclusion of gray literature, lack of qualitative research, and a search strategy that used a limited number of academic databases means it is possible the review has not included all studies relevant to VR relaxation for people with mental health conditions and, therefore, there may be additional evidence excluded from this review. Given that the EPHPP tool reclassifies RCTs as CCTs if they do not adequately describe the randomization method, regardless of whether the published paper describes itself as an RCT, it could be argued that the review underreports the number of RCTs in this area of research. Perceived limited information on certain domains, e.g., confounders, by researchers may also have impacted on quality ratings of studies.

### Clinical applications

A previous systematic review on VR relaxation for the general population highlighted its possibility as a low-intensity intervention for people with mental health conditions [15]. This review is consistent with that finding and shows that most of the evidence for VR relaxation as a clinical intervention is in anxiety and stress-related problems, suggesting the possibility of piloting the VR relaxation in anxiety clinics; but the findings also suggest the potential of VR relaxation for service users with a wider range of mental health diagnoses. Given that stress is a major trigger and ongoing problem in a range of mental health conditions, including for people with severe mental health conditions on psychiatric wards, its application may be wider, as indicated by some of the inpatient studies included in this review.

There is now considerable research integrating VR with cognitive behavior therapies [12, 44]. There may be ways of integrating VR relaxation with cognitive behavioral therapy, as a behavioral means of coping with stress. In cognitive behavioral interventions that use VR relaxation, it may be helpful to work with service users to develop real-world applications for their relaxation practices that build on the experiential learning gained in the VR, as a way of creating a treatment that has sustainability and is tailored to individuals [45]. However, it is also important to recognize that VR relaxation has potential as a standalone intervention, that can stand apart from other psychosocial interventions, and gives people with mental health problems the same access to relaxation as the general population.

The increased availability, accessibility, and portability of VR indicates that VR relaxation may be a useful method of prevention, management, and treatment of mental health problems outside of the clinic, including at the homes and workplaces of people with mental health problems [15, 46, 47]. It could be particularly beneficial for those who lack access to real-world nature, such as people living in urban areas or those with mobility difficulties. Some caution to this interpretation might need to be applied to conditions like social anxiety, where use of home-based VR relaxation as a coping strategy might reduce beneficial exposure to real-world social situations, and inadvertently function as a maintenance factor or safety behaviour, potentially worsening symptoms [48]. Nevertheless, there are people with mental health conditions who can access real-world nature but do not do so because of their mental health problems, and VR relaxation may be a way to overcome this problem and make relaxation easier.

### Future research

Future research should prioritize longitudinal studies to assess if there are sustained, longer-term benefits of VR relaxation for people with mental health conditions. These studies should aim to provide greater standardization of interventions, definitions, and measures; recruit larger numbers from more diverse populations, including a wider range of mental health conditions, and a variety of age groups, including young people and older adults, to aid generalisability. Mixed diagnosis samples powered adequately to perform subgroup analysis could be a useful dimension to this research. Service users should be involved in this future research and have a key role in development of VR relaxation interventions. A key next step would be to evaluate VR relaxation for people with mental health conditions in more naturalistic clinical and non-clinical settings, such as clinics, psychiatric wards, or in the home. These settings would provide further evidence of VR relaxation as a protective measure to reduce the strain on mental health services, especially given that existing studies are mainly conducted in laboratory settings. Future reviews of the literature might also consider using specific theoretical models of technology to provide a more in-depth analysis of the research.

Given the dominance of nature-based scenes in the environments, it would be useful to evaluate the importance of HMDs by comparing VR-based nature with similar scenes on 2D screens, including on mobile devices, particularly given the ultra-high-definition quality of many 2D screen projections, as this may provide a different kind of immersive user experience. Although 2D interventions are likely to be less immersive and experiential and may not hold users’ attention so effectively, they may still promote relaxation and reduce stress for people with mental health conditions. This may be one way to make these benefits available to a wider audience, potentially using technology that is likely to be more easily accessed or already possessed by many mental health service users.

## Conclusion

This is the first systematic review to narratively synthesize current evidence on VR relaxation for people with mental health conditions. All studies used nature-based VR environments and indicated that they are feasible, acceptable, and effective in the short term to promote relaxation and reduce stress, especially for people with anxiety disorders and stress-related problems. Despite methodological limitations, this appears to be a promising intervention for people with mental health conditions and an important area of future research.

## Supplementary Information

Below is the link to the electronic supplementary material.

Supplementary file1 (DOCX 21 KB)

## Data Availability

Data sharing not applicable to this article.
