# Supplementary material for: Virtual reality relaxation for people with mental health conditions: a systematic review
Source: Soc Psychiatry Psychiatr Epidemiol. 2023 Jan 20;58(7):989–1007. doi: 10.1007/s00127-022-02417-5 (PMC9852806; doi:10.1007/s00127-022-02417-5)
Supplement: Supplementary file 1 — Supplementary file1 (DOCX 21 KB) [file 127_2022_2417_MOESM1_ESM.docx]

**Supplementary 1. Table 3.** Full breakdown of quality ratings using the Effective Public Health Practice Project of studies of virtual reality relaxation for people with mental health conditions

| Study | A. Selection bias  Q1. Are individuals selected to participate in the study likely to represent the target population? Very likely = 1, Somewhat likely = 2, Not likely = 3, Can’t tell = 4.  Q2. What percentage of selected individuals agreed to participate? 80-100% agreement = 1, 60-79% agreement = 2, less than 60% agreement = 3, Not applicable = 4, Can’t tell = 5. | B. Study design  Q1. Study design  Randomised controlled trial = 1, controlled clinical trial = 2, cohort analytic = 3, case-control = 4, cohort = 5, interrupted time series = 6, other = 7, Can’t tell = 8. Was the study described as randomised? If yes, was the method of randomisation described? Was the method appropriate? | C. Confounders  Q1. Were there important differences between groups prior to the intervention? Yes = 1, No = 2, Can’t tell = 3. Examples of confounders: race, sex, marital status/family, age, socio-economic status, education, health status, pre-intervention score on outcome measures.  Q2.  Percentage of relevant confounders that were controlled. 80-100% (most) = 1, 60-79% (some) = 2, less than 60% (few or more) = 3, Can’t tell = 4. | D. Blinding  Q1. Was the outcome assessor aware of the intervention or exposure status of participants? Yes = 1, No = 2, Can’t tell = 3.  Q2. Were the study participants aware of the research questions? Yes = 1, No = 2, Can’t tell = 3. | E. Data collection method  Q1. Were data collection tools shown to be valid? Yes = 1, No = 2, Can’t tell = 3.  Q2. Were data collection tools shown to be reliable? Yes = 1, No = 2, Can’t tell = 3. | F. Withdrawals and drop-outs  Q1. Were withdrawals and drop-outs reported in terms of numbers and/or reasons per group? Yes = 1, No = 2, Can’t tell = 3, Not applicable (i.e., one-time surveys or interviews) = 4.  Q2. Percentage of participants completing the study. 80-100% = 1, 60-79% = 2, less than 60% = 3, Can’t tell = 4, Not applicable (i.e., retrospective case-control) = 5. | Global rating |
| --- | --- | --- | --- | --- | --- | --- | --- |
| Bossenbroek et al (2020) | (Q1) 2. Somewhat likely; (Q2) 1. 80-100% agreement = MODERATE | (Q1) 5. Cohort = MODERATE | (Q1) 2. No  (Q2) Not Applicable = STRONG | (Q1) 3. Can’t tell; (Q2) 3. Can’t tell = MODERATE | (Q1) 1. Yes; (Q2) 1. Yes = STRONG | (Q1) 1. Yes; (Q2) 1. 80-100% = STRONG | STRONG |
| Gorini et al (2010) | (Q1) 2. Somewhat likely; (Q2) 5. Can’t tell = MODERATE | (Q1) 2. Controlled clinical trial = STRONG | (Q1) 3. Can’t tell; (Q2) 4. Can’t tell = WEAK | (Q1) 3. Can’t tell; (Q2) 3. Can’t tell = MODERATE | (Q1) 1. Yes; (Q2) 3. Can’t tell = MODERATE | (Q1) 2. No; (Q2) 3. Can’t tell = WEAK | WEAK |
| Habak et al (2021) | (Q1) 2. Somewhat likely; (Q2) 1. 80-100% agreement = MODERATE | (Q1) 5. Cohort = MODERATE | (Q1) 2. No  (Q2) Not Applicable = STRONG | (Q1) 3. Can’t tell; (Q2) 3. Can’t tell = MODERATE | (Q1) 1. Yes; (Q2) 3. Can’t tell = MODERATE | (Q1) 1. Yes; (Q2) 1. 80-100% = STRONG | STRONG |
| Kim et al (2021) | (Q1) 2. Somewhat likely; (Q2) 5. Can’t tell = MODERATE | (Q1) 2. Controlled clinical trial = STRONG | (Q1) 3. Can’t tell; (Q2) 4. Can’t tell = WEAK | (Q1) 3. Can’t tell; (Q2) 4. Can’t tell = MODERATE | (Q1) 1. Yes; (Q2) 3. Can’t tell = MODERATE | (Q1) 1. Yes; (Q2) 1. 80-100% = STRONG | MODERATE |
| Maarsingh et al (2019) | (Q1) 2. Somewhat likely; (Q2) 5. Can’t tell = MODERATE | (Q1) 4. Case-Control = MODERATE | (Q1) 3. Can’t tell; (Q2) 4. Can’t tell = WEAK | (Q1) 3. Can’t tell; (Q2) 4. Can’t tell = MODERATE | (Q1) 1. Yes; (Q2) 1. Yes = STRONG | (Q1) 1. Yes; (Q2) 3. Less than 60% = WEAK | WEAK |
| Malbos et al (2020) | (Q1) 2. Somewhat likely; (Q2) 2. 60-79% agreement = MODERATE | (Q1) 1. Randomised Control Trial = STRONG | (Q1) 3. Can’t tell; (Q2) 4. Can’t tell = WEAK | (Q1) 3. Can’t tell; (Q2) 3. Can’t tell = MODERATE | (Q1) 1. Yes; (Q2) 3. Can’t tell = MODERATE | (Q1) 1. Yes; (Q2) 2. 60-79% = MODERATE | MODERATE |
| Manzoni et al (2008) | (Q1) 2. Somewhat likely; (Q2) 1. 80-100% agreement = MODERATE | (Q1) 1. Randomised controlled trial = STRONG | (Q1) 2. No; (Q2) Not Applicable = STRONG | (Q1) 3. Can’t tell; (Q2) 3. Can’t tell = MODERATE | (Q1) 1. Yes; (Q2) 3. Can’t tell = MODERATE | (Q1) 1. Yes; (Q2) 1. 80-100% = STRONG | STRONG |
| Manzoni et al (2009) | (Q1) 2. Somewhat likely; (Q2) 2. 60-79% agreement = MODERATE | (Q1) 1. Randomised controlled trial = STRONG | (Q1) 2. No; (Q2) Not Applicable = STRONG | (Q1) 3. Can’t tell; (Q2) 3. Can’t tell = MODERATE | (Q1) 1. Yes; (Q2) 3. Can’t tell = MODERATE | (Q1) 1. Yes; (Q2) 2. 60-79% = MODERATE | STRONG |
| Mark et al (2021) | (Q1) 2. Somewhat likely; (Q2) 2. 60-79% agreement = MODERATE | (Q1) 5. Cohort = MODERATE | (Q1) 2. No  (Q2) Not Applicable = STRONG | (Q1) 3. Can’t tell; (Q2) 3. Can’t tell = MODERATE | (Q1) 3. Can’t tell; (Q2) 3. Can’t tell = WEAK | (Q1) 1. Yes; (Q2) 1. 80-100% = STRONG | MODERATE |
| Mistry et al (2020) | (Q1) 2. Somewhat likely; (Q2) 5. Can’t tell = MODERATE | (Q1) 2. Controlled clinical trial = STRONG | (Q1) 1. Yes;  (Q2) 4. Can’t tell = WEAK | (Q1) 3. Can’t tell; (Q2) 3. Can’t tell = MODERATE | (Q1) 1. Yes; (Q2) 3. Can’t tell = MODERATE | (Q1) 1. Yes; (Q2) 1. 80-100% = STRONG | MODERATE |
| Pallavicini et al (2009) | (Q1) 2. Somewhat likely; (Q2) 1. 80-100% = MODERATE | (Q1) 1. Randomised controlled trial = STRONG | (Q1) 2. No; (Q2) Not Applicable = STRONG | (Q1) 3. Can’t tell; (Q2) 3. Can’t tell = MODERATE | (Q1) 1. Yes; (Q2) 3. Can’t tell = MODERATE | (Q1) 1. Yes; (Q2) 1. 80-100% = STRONG | STRONG |
| Repetto et al (2013) | (Q1) 2. Somewhat likely; (Q2) 1. 80-100% = MODERATE | (Q1) 1. Randomised controlled trial = STRONG | (Q1) 2. No; (Q2) Not Applicable = STRONG | (Q1) 3. Can’t tell; (Q2) 3. Can’t tell = MODERATE | (Q1) 1. Yes; (Q2) 3. Can’t tell = MODERATE | (Q1) 1. Yes; (Q2) 1. 80-100% = STRONG | STRONG |
| Riva et al (2008) | (Q1) 2. Somewhat likely; (Q2) 5. Can’t tell = MODERATE | (Q1) 2. Controlled clinical trial= STRONG | (Q1) 3. Can’t tell; (Q2) 4. Can’t tell = WEAK | (Q1) 3. Can’t tell; (Q2) 3. Can’t tell = MODERATE | (Q1) 3. Can’t tell; (Q2) 3. Can’t tell = WEAK | (Q1) 2. No; (Q2) 3. Can’t tell = WEAK | WEAK |
| Shah et al (2015) | (Q1) 2. Somewhat likely; (Q2) 1. 80-100% agreement = MODERATE | (Q1) 5. Cohort = MODERATE | (Q1) 1. No; (Q2) . Not Applicable = STRONG | (Q1) 3. Can’t tell; (Q2) 3. Can’t tell = MODERATE | (Q1) 1. Yes; (Q2) 1. Yes = STRONG | (Q1) 1. Yes; (Q2) 1. 80-100% = STRONG | STRONG |
| Tan et al (2021) | (Q1) 2. Somewhat likely; (Q2) 2. 60-79% agreement = MODERATE | (Q1) 1. Randomised controlled trial = STRONG | (Q1) 2. Yes; (Q2) 1. 80-100% = STRONG | (Q1) 3. Can’t tell; (Q2) 3. Can’t tell = MODERATE | (Q1) 1. Yes; (Q2) 1. Yes = STRONG | (Q1) 1. Yes; (Q2) 1. 80-100% = STRONG | STRONG |
| Tarrant et al (2018) | (Q1) 2. Somewhat likely; (Q2) 2. 60-79% agreement = MODERATE | (Q1) 2. Controlled clinical trial = STRONG | (Q1) 1. No; (Q2) Not Applicable = STRONG | (Q1) 3. Can’t tell; (Q2) 3. Can’t tell = MODERATE | (Q1) 1. Yes; (Q2) 1. Yes = STRONG | (Q1) 1. Yes; (Q2) 2. 60-79% = MODERATE | STRONG |
| Veling et al (2021) | (Q1) 2. Somewhat likely; (Q2) 2. 60-79% agreement = MODERATE | (Q1) 1. Randomised controlled trial = STRONG | (Q1) 1. No; (Q2) Not Applicable = STRONG | (Q1) 3. Can’t tell; (Q2) 3. Can’t tell = MODERATE | (Q1) 1. Yes; (Q2) 1. Yes = STRONG | (Q1) 1. Yes; (Q2) 1. 80-100% = STRONG | STRONG |
| Wang et al (2020) | (Q1) 2. Somewhat likely; (Q2) 1. 80-100% agreement = MODERATE | (Q1) 2. Controlled clinical trial= STRONG | Q1) 1. No ; (Q2) Not Applicable = STRONG | (Q1) 3. Can’t tell; (Q2) 3. Can’t tell = MODERATE | (Q1) 1. Yes; (Q2) 1. Yes = STRONG | (Q1) 1. Yes; (Q2) 1. 80-100% = STRONG | STRONG |

**Supplementary 2.** Search strategy used to extract studies investigating virtual reality relaxation for people with mental health conditions

APA PsycInfo <1806 to September Week 2 2021>

1 exp virtual reality/ 9781

2 virtual real*.mp. [mp=title, abstract, heading word, table of contents, key concepts, original title, tests & measures, mesh] 11481

3 virtual-real*.mp. [mp=title, abstract, heading word, table of contents, key concepts, original title, tests & measures, mesh] 11481

4 VR.mp. [mp=title, abstract, heading word, table of contents, key concepts, original title, tests & measures, mesh] 4007

5 virtual enviro*.mp. [mp=title, abstract, heading word, table of contents, key concepts, original title, tests & measures, mesh] 4377

6 virtual character*.mp. [mp=title, abstract, heading word, table of contents, key concepts, original title, tests & measures, mesh] 209

7 VCs.mp. [mp=title, abstract, heading word, table of contents, key concepts, original title, tests & measures, mesh] 265

8 avatar*.mp. [mp=title, abstract, heading word, table of contents, key concepts, original title, tests & measures, mesh] 1876

9 1 or 2 or 3 or 4 or 5 or 6 or 7 or 8 16124

10 exp Relaxation/ 2582

11 relax*.mp. [mp=title, abstract, heading word, table of contents, key concepts, original title, tests & measures, mesh] 24730

12 autogen*.mp. [mp=title, abstract, heading word, table of contents, key concepts, original title, tests & measures, mesh] 1435

13 meditat*.mp. [mp=title, abstract, heading word, table of contents, key concepts, original title, tests & measures, mesh] 10813

14 mindful*.mp. [mp=title, abstract, heading word, table of contents, key concepts, original title, tests & measures, mesh] 19838

15 rest*.mp. [mp=title, abstract, heading word, table of contents, key concepts, original title, tests & measures, mesh] 192448

16 PMR.mp. [mp=title, abstract, heading word, table of contents, key concepts, original title, tests & measures, mesh] 330

17 progressive muscle.mp. [mp=title, abstract, heading word, table of contents, key concepts, original title, tests & measures, mesh] 866

18 imagery.mp. [mp=title, abstract, heading word, table of contents, key concepts, original title, tests & measures, mesh] 29491

19 breath*.mp. [mp=title, abstract, heading word, table of contents, key concepts, original title, tests & measures, mesh] 14532

20 distract*.mp. [mp=title, abstract, heading word, table of contents, key concepts, original title, tests & measures, mesh] 26857

21 wellness.mp. [mp=title, abstract, heading word, table of contents, key concepts, original title, tests & measures, mesh] 8636

22 wellbeing.mp. [mp=title, abstract, heading word, table of contents, key concepts, original title, tests & measures, mesh] 16804

23 well-being.mp. [mp=title, abstract, heading word, table of contents, key concepts, original title, tests & measures, mesh] 102769

24 10 or 11 or 12 or 13 or 14 or 15 or 16 or 17 or 18 or 19 or 20 or 21 or 22 or 23 409659

25 exp Mental Disorders/ 903120

26 mental health.mp. [mp=title, abstract, heading word, table of contents, key concepts, original title, tests & measures, mesh] 240022

27 mental illness.mp. [mp=title, abstract, heading word, table of contents, key concepts, original title, tests & measures, mesh] 47072

28 mental disorder.mp. [mp=title, abstract, heading word, table of contents, key concepts, original title, tests & measures, mesh] 12601

29 psych*.mp. [mp=title, abstract, heading word, table of contents, key concepts, original title, tests & measures, mesh] 1621760

30 schiz*.mp. [mp=title, abstract, heading word, table of contents, key concepts, original title, tests & measures, mesh] 149173

31 mood.mp. [mp=title, abstract, heading word, table of contents, key concepts, original title, tests & measures, mesh] 84172

32 depress*.mp. [mp=title, abstract, heading word, table of contents, key concepts, original title, tests & measures, mesh] 392491

33 bipolar.mp. [mp=title, abstract, heading word, table of contents, key concepts, original title, tests & measures, mesh] 44966

34 anxi*.mp. [mp=title, abstract, heading word, table of contents, key concepts, original title, tests & measures, mesh] 269302

35 panic disorder.mp. [mp=title, abstract, heading word, table of contents, key concepts, original title, tests & measures, mesh] 12749

36 obsessive compulsive.mp. [mp=title, abstract, heading word, table of contents, key concepts, original title, tests & measures, mesh] 25049

37 obsessive-compulsive.mp. [mp=title, abstract, heading word, table of contents, key concepts, original title, tests & measures, mesh] 25049

38 OCD.mp. [mp=title, abstract, heading word, table of contents, key concepts, original title, tests & measures, mesh] 11626

39 stress.mp. [mp=title, abstract, heading word, table of contents, key concepts, original title, tests & measures, mesh] 267586

40 PTSD.mp. [mp=title, abstract, heading word, table of contents, key concepts, original title, tests & measures, mesh] 39759

41 dissociat*.mp. [mp=title, abstract, heading word, table of contents, key concepts, original title, tests & measures, mesh] 34709

42 eating.mp. [mp=title, abstract, heading word, table of contents, key concepts, original title, tests & measures, mesh] 67920

43 anorexi*.mp. [mp=title, abstract, heading word, table of contents, key concepts, original title, tests & measures, mesh] 19013

44 bulim*.mp. [mp=title, abstract, heading word, table of contents, key concepts, original title, tests & measures, mesh] 13943

45 substance us*.mp. [mp=title, abstract, heading word, table of contents, key concepts, original title, tests & measures, mesh] 68367

46 substance misuse.mp. [mp=title, abstract, heading word, table of contents, key concepts, original title, tests & measures, mesh] 3446

47 addict*.mp. [mp=title, abstract, heading word, table of contents, key concepts, original title, tests & measures, mesh] 66457

48 dissocial.mp. [mp=title, abstract, heading word, table of contents, key concepts, original title, tests & measures, mesh] 291

49 personality disorder.mp. [mp=title, abstract, heading word, table of contents, key concepts, original title, tests & measures, mesh] 31946

50 25 or 26 or 27 or 28 or 29 or 30 or 31 or 32 or 33 or 34 or 35 or 36 or 37 or 38 or 39 or 40 or 41 or 42 or 43 or 44 or 45 or 46 or 47 or 48 or 49 2442519

51 9 and 24 and 50 806

Embase <1974 to 2021 Week 36>

1 exp virtual reality/ 20291

2 virtual real*.mp. [mp=title, abstract, heading word, drug trade name, original title, device manufacturer, drug manufacturer, device trade name, keyword heading word, floating subheading word, candidate term word] 25760

3 virtual-real*.mp. [mp=title, abstract, heading word, drug trade name, original title, device manufacturer, drug manufacturer, device trade name, keyword heading word, floating subheading word, candidate term word] 25760

4 VR.mp. [mp=title, abstract, heading word, drug trade name, original title, device manufacturer, drug manufacturer, device trade name, keyword heading word, floating subheading word, candidate term word] 14342

5 virtual enviro*.mp. [mp=title, abstract, heading word, drug trade name, original title, device manufacturer, drug manufacturer, device trade name, keyword heading word, floating subheading word, candidate term word] 4516

6 virtual character*.mp. [mp=title, abstract, heading word, drug trade name, original title, device manufacturer, drug manufacturer, device trade name, keyword heading word, floating subheading word, candidate term word] 174

7 VCs.mp. [mp=title, abstract, heading word, drug trade name, original title, device manufacturer, drug manufacturer, device trade name, keyword heading word, floating subheading word, candidate term word] 1578

8 avatar*.mp. [mp=title, abstract, heading word, drug trade name, original title, device manufacturer, drug manufacturer, device trade name, keyword heading word, floating subheading word, candidate term word] 2163

9 1 or 2 or 3 or 4 or 5 or 6 or 7 or 8 39271

10 exp Relaxation/ 39070

11 relax*.mp. [mp=title, abstract, heading word, drug trade name, original title, device manufacturer, drug manufacturer, device trade name, keyword heading word, floating subheading word, candidate term word] 223285

12 autogen*.mp. [mp=title, abstract, heading word, drug trade name, original title, device manufacturer, drug manufacturer, device trade name, keyword heading word, floating subheading word, candidate term word] 18050

13 meditat*.mp. [mp=title, abstract, heading word, drug trade name, original title, device manufacturer, drug manufacturer, device trade name, keyword heading word, floating subheading word, candidate term word] 12847

14 mindful*.mp. [mp=title, abstract, heading word, drug trade name, original title, device manufacturer, drug manufacturer, device trade name, keyword heading word, floating subheading word, candidate term word] 18592

15 rest*.mp. [mp=title, abstract, heading word, drug trade name, original title, device manufacturer, drug manufacturer, device trade name, keyword heading word, floating subheading word, candidate term word] 1731372

16 PMR.mp. [mp=title, abstract, heading word, drug trade name, original title, device manufacturer, drug manufacturer, device trade name, keyword heading word, floating subheading word, candidate term word] 5466

17 progressive muscle.mp. [mp=title, abstract, heading word, drug trade name, original title, device manufacturer, drug manufacturer, device trade name, keyword heading word, floating subheading word, candidate term word] 3896

18 imagery.mp. [mp=title, abstract, heading word, drug trade name, original title, device manufacturer, drug manufacturer, device trade name, keyword heading word, floating subheading word, candidate term word] 23649

19 breath*.mp. [mp=title, abstract, heading word, drug trade name, original title, device manufacturer, drug manufacturer, device trade name, keyword heading word, floating subheading word, candidate term word] 349019

20 distract*.mp. [mp=title, abstract, heading word, drug trade name, original title, device manufacturer, drug manufacturer, device trade name, keyword heading word, floating subheading word, candidate term word] 41661

21 wellness.mp. [mp=title, abstract, heading word, drug trade name, original title, device manufacturer, drug manufacturer, device trade name, keyword heading word, floating subheading word, candidate term word] 16636

22 wellbeing.mp. [mp=title, abstract, heading word, drug trade name, original title, device manufacturer, drug manufacturer, device trade name, keyword heading word, floating subheading word, candidate term word] 90395

23 well-being.mp. [mp=title, abstract, heading word, drug trade name, original title, device manufacturer, drug manufacturer, device trade name, keyword heading word, floating subheading word, candidate term word] 126731

24 10 or 11 or 12 or 13 or 14 or 15 or 16 or 17 or 18 or 19 or 20 or 21 or 22 or 23 2528212

25 exp Mental Disorders/ 2335678

26 mental health.mp. [mp=title, abstract, heading word, drug trade name, original title, device manufacturer, drug manufacturer, device trade name, keyword heading word, floating subheading word, candidate term word] 325309

27 mental illness.mp. [mp=title, abstract, heading word, drug trade name, original title, device manufacturer, drug manufacturer, device trade name, keyword heading word, floating subheading word, candidate term word] 42124

28 mental disorder.mp. [mp=title, abstract, heading word, drug trade name, original title, device manufacturer, drug manufacturer, device trade name, keyword heading word, floating subheading word, candidate term word] 13718

29 psych*.mp. [mp=title, abstract, heading word, drug trade name, original title, device manufacturer, drug manufacturer, device trade name, keyword heading word, floating subheading word, candidate term word] 1979334

30 schiz*.mp. [mp=title, abstract, heading word, drug trade name, original title, device manufacturer, drug manufacturer, device trade name, keyword heading word, floating subheading word, candidate term word] 243815

31 mood.mp. [mp=title, abstract, heading word, drug trade name, original title, device manufacturer, drug manufacturer, device trade name, keyword heading word, floating subheading word, candidate term word] 155616

32 depress*.mp. [mp=title, abstract, heading word, drug trade name, original title, device manufacturer, drug manufacturer, device trade name, keyword heading word, floating subheading word, candidate term word] 848566

33 bipolar.mp. [mp=title, abstract, heading word, drug trade name, original title, device manufacturer, drug manufacturer, device trade name, keyword heading word, floating subheading word, candidate term word] 119405

34 anxi*.mp. [mp=title, abstract, heading word, drug trade name, original title, device manufacturer, drug manufacturer, device trade name, keyword heading word, floating subheading word, candidate term word] 436748

35 panic disorder.mp. [mp=title, abstract, heading word, drug trade name, original title, device manufacturer, drug manufacturer, device trade name, keyword heading word, floating subheading word, candidate term word] 12450

36 obsessive compulsive.mp. [mp=title, abstract, heading word, drug trade name, original title, device manufacturer, drug manufacturer, device trade name, keyword heading word, floating subheading word, candidate term word] 35071

37 obsessive-compulsive.mp. [mp=title, abstract, heading word, drug trade name, original title, device manufacturer, drug manufacturer, device trade name, keyword heading word, floating subheading word, candidate term word] 35071

38 OCD.mp. [mp=title, abstract, heading word, drug trade name, original title, device manufacturer, drug manufacturer, device trade name, keyword heading word, floating subheading word, candidate term word] 15480

39 stress.mp. [mp=title, abstract, heading word, drug trade name, original title, device manufacturer, drug manufacturer, device trade name, keyword heading word, floating subheading word, candidate term word] 1388605

40 PTSD.mp. [mp=title, abstract, heading word, drug trade name, original title, device manufacturer, drug manufacturer, device trade name, keyword heading word, floating subheading word, candidate term word] 37286

41 dissociat*.mp. [mp=title, abstract, heading word, drug trade name, original title, device manufacturer, drug manufacturer, device trade name, keyword heading word, floating subheading word, candidate term word] 186831

42 eating.mp. [mp=title, abstract, heading word, drug trade name, original title, device manufacturer, drug manufacturer, device trade name, keyword heading word, floating subheading word, candidate term word] 138474

43 anorexi*.mp. [mp=title, abstract, heading word, drug trade name, original title, device manufacturer, drug manufacturer, device trade name, keyword heading word, floating subheading word, candidate term word] 100807

44 bulim*.mp. [mp=title, abstract, heading word, drug trade name, original title, device manufacturer, drug manufacturer, device trade name, keyword heading word, floating subheading word, candidate term word] 16847

45 substance us*.mp. [mp=title, abstract, heading word, drug trade name, original title, device manufacturer, drug manufacturer, device trade name, keyword heading word, floating subheading word, candidate term word] 57815

46 substance misuse.mp. [mp=title, abstract, heading word, drug trade name, original title, device manufacturer, drug manufacturer, device trade name, keyword heading word, floating subheading word, candidate term word] 4222

47 addict*.mp. [mp=title, abstract, heading word, drug trade name, original title, device manufacturer, drug manufacturer, device trade name, keyword heading word, floating subheading word, candidate term word] 155201

48 dissocial.mp. [mp=title, abstract, heading word, drug trade name, original title, device manufacturer, drug manufacturer, device trade name, keyword heading word, floating subheading word, candidate term word] 304

49 personality disorder.mp. [mp=title, abstract, heading word, drug trade name, original title, device manufacturer, drug manufacturer, device trade name, keyword heading word, floating subheading word, candidate term word] 49294

50 25 or 26 or 27 or 28 or 29 or 30 or 31 or 32 or 33 or 34 or 35 or 36 or 37 or 38 or 39 or 40 or 41 or 42 or 43 or 44 or 45 or 46 or 47 or 48 or 49 5231834

51 9 and 24 and 50 1679
